# Influence of sealer type on treatment outcome of teeth with apical periodontitis: a systematic review

**DOI:** 10.1590/0103-6440202305471

**Published:** 2023-12-22

**Authors:** Pablo Amoroso-Silva, Sabrina C. Brasil, Alejandro R. Pérez, Elen S. Tolentino, Flávio R. F. Alves, José F. Siqueira, Isabela N. Rôças

**Affiliations:** 1 Postgraduate Program in Dentistry, University of Grande Rio (UNIGRANRIO), Rio de Janeiro, RJ, Brazil.; 2 Department of Endodontics, University Rey Juan Carlos, Madrid, Spain; 3 Department of Dentistry, State University of Maringá, Maringá, PR, Brazil.; 4 Department of Dental Research, Faculty of Dentistry, Iguaçu University (UNIG), Nova Iguaçu, RJ, Brazil.

**Keywords:** apical periodontitis, root canal sealers, obturation materials, root canal filling

## Abstract

The purpose of this article was to Evaluate the influence of sealer on the outcome of non-surgical endodontic treatment or retreatment of permanent teeth with apical periodontitis (PROSPERO registration: CRD42020205951).

**Methodology****:**

A systematic review of original clinical studies was carried out following PRISMA guidelines to answer whether the type of sealer used in endodontic treatment or retreatment influences the repair of apical periodontitis determined by clinical and radiographic parameters. Electronic searches were performed in PubMed, Embase, Web of Science, Scopus, and the Cochrane Library database, until May 2023. Gray literature and a hand search of reference lists were also performed. The risk of bias was assessed using Cochrane RoB2 for randomized trials and the Newcastle-Ottawa Scale (NOS) for prospective and retrospective cohort and case-control studies.

**Results****:**

Among 1046 studies, a total of 819 were selected by title and abstract, resulting in 23 for full-text review. In total, 11 studies met the inclusion criteria (1467 patients/teeth with apical periodontitis). The quality assessment using RoB2 included five randomized control trials, of which four had medium risk and one had a low risk of bias. According to the NOS scale, five studies were classified as low risk and one study was considered as medium risk of bias. The sealer type and obturation techniques varied, and the mean follow-up time was 3.7 years. Most studies used two-dimensional radiographic criteria to assess the treatment outcome sealers and not cements. Eight studies did not find significant differences when comparing cements. The healing rates ranged from 56.7% to 90%.

**Conclusions****:**

The results of this review support that the current endodontic sealers do not seem to influence the treatment outcome of permanent teeth with apical periodontitis. Although the studies had medium and low risk of bias, the results should be interpreted with caution. More randomized studies of long-term outcomes comparing filling materials are needed to strengthen this claim and allow for a meta-analysis.

## Resumo

O objetivo deste artigo foi avaliar a influência do cimento no resultado do tratamento ou retratamento endodôntico não cirúrgico de dentes permanentes com periodontite apical (registro PROSPERO: CRD42020205951).

**Metodologia****:**

Uma revisão sistemática de estudos clínicos originais foi realizada seguindo as diretrizes PRISMA para responder se o tipo de cimento usado no tratamento ou retratamento endodôntico influencia a reparação da periodontite apical determinada por parâmetros clínicos e radiográficos. Pesquisas eletrônicas foram realizadas no PubMed, Embase, Web of Science, Scopus e no banco de dados Cochrane Library, até maio de 2023. A literatura cinza e uma pesquisa manual das listas de referências também foram realizadas. O risco de viés foi avaliado usando Cochrane RoB2 para os estudos randomizados e Newcastle-Ottawa Scale (NOS) para coorte prospectiva e retrospectiva e estudos de caso-controle.

**Resultados:**

Entre 1.046 estudos, um total de 819 foram selecionados por título e resumo, resultando em 23 para revisão de texto completo. No total, 11 estudos preencheram os critérios de inclusão (1.467 pacientes/dentes com periodontite apical). A avaliação de qualidade usando RoB2 incluiu cinco estudos randomizados de controle, dos quais quatro tinham risco médio e um tinha baixo risco de viés. De acordo com a escala NOS, cinco estudos foram classificados com baixo risco e um estudo foi considerado com médio risco de viés. O tipo de cimento e as técnicas de obturação variaram, e o tempo médio de acompanhamento foi de 3,7 anos. A maioria dos estudos utilizou critérios radiográficos bidimensionais para avaliar o resultado do tratamento. Oito estudos não encontraram diferenças significativas ao comparar os cimentos. As taxas de reparação variaram de 56,7% a 90%.

**Conclusões****:**

Os resultados desta revisão suportam que os cimentos endodônticos atuais não parecem influenciar o resultado do tratamento de dentes permanentes com periodontite apical. Embora os estudos tiveram médio e baixo risco de viés, os resultados devem ser interpretados com cautela. Mais estudos randomizados de resultados de longo prazo comparando materiais de obturação são necessários para fortalecer essa afirmação e permitir uma meta-análise.

## Introduction

The main objectives of root canal obturation are to prevent infection or reinfection of the root canal system and negate access of residual bacteria to the periradicular tissues ^1^). Filling materials can be classified as core materials and sealers; the former occupy the major volume of the prepared canal, while the latter are used to fill not only the space between dentinal canal walls and the core material, but also other irregularities and complexities of the canal system. Sealers are generally classified as zinc oxide-eugenol-based, calcium hydroxide-containing, resinous, silicone-based, glass ionomer, and calcium silicate (or bioceramic) sealers. Because core materials are physically unable to reach and fill all areas and irregularities of the root canal system and have no ability to adhere to dentin, sealers play an essential role in promoting an adequate hydraulic and antibacterial seal ^2^ ^),(^ ^3^ ^),(^ ^4^. 

Apical periodontitis is a highly prevalent disease in adult individuals ^5^ and the most conservative approach to treat this condition is nonsurgical endodontic treatment ^6^. The success rate in teeth with apical periodontitis is significantly reduced when compared to teeth with no preoperative lesions ^7^. Complete disinfection and subsequent sealing of the root canal system are essential for achieving endodontic success ^6^.

Earlier formaldehyde-containing sealers were developed with the purpose of fixating pulp tissue remnants and providing antimicrobial properties, but they have been demonstrated to be highly cytotoxic and compromise the periradicular tissue healing response when extruded ^11^ ^), (^ ^12^. For the large majority of current root canal sealers cytotoxicity and antibacterial activities are observed following mixing and throughout the setting process ^13^ ^),(^ ^14^ ^),^ but are mostly lost or significantly reduced when setting is complete ^15^ ^),(^ ^16^.

Studies have shown a lower healing rate in teeth with overfillings ^9^ ^),(^ ^17^ ^),^ raising the possibility that this could be a result of the filling material cytotoxicity or a foreign-body reaction ^18^ ^),(^ ^19^ ^),(^ ^21^ ^),(^ ^22^. Therefore, the root canal sealer might have a biological influence on the treatment outcome. Several properties of these materials may potentially interfere with treatment outcomes, especially biocompatibility, sealing ability and antibacterial activity. 

Outcome studies in humans comparing different filling materials are of great relevance to determine if they influence the result of root canal treatment. The aim of this systematic review is to evaluate the influence of root canal sealers on healing of apical periodontitis, as determined by radiographic and clinical parameters. 

## Material and methods

A systematic review of the best evidence available in the literature was performed following the most recent recommendations of to the Preferred Reporting Items for Systematic Reviews and Meta-Analyses (PRISMA) guidelines (http://www.prisma-statement.org) ^23^. The review was registered on the international database of prospectively registered systematic reviews with a health-related outcome (PROSPERO) under number CRD4202020595. 

### Review question

The focused question was: ‘In patients/teeth with apical periodontitis what is the influence of the type of sealer in root canal treatment or retreatment in terms of clinical and radiographic-related outcomes?’. The following PICOT strategy was used: (P) patients/permanent teeth with apical periodontitis subjected to root canal treatment or retreatment with (I) any root canal sealer in comparison with (C) any other root canal sealer and addressing (O) clinical and radiographic outcomes for (T) at least one-year post-op. 

### Eligibility criteria

- *Studies:* original clinical studies based on humans (randomized controlled clinical trials, nonrandomized clinical trials, retrospective and prospective comparative cohort and case-control studies);
- *Participants and intervention:* patients with apical periodontitis who have undergone root canal treatment or retreatment in permanent teeth, not subjected to periradicular surgery;
- *Comparison:* studies that addressed root canal treatment in permanent teeth and compared two or more sealers;
- *Outcome:* outcomes were a combination of clinical and radiographic outcome measures. The most critical outcomes were ‘success rate’ and radiographic evidence of apical healing. Other outcomes included clinical absence of signs and symptoms (such as pain, tenderness, swelling, need for medication)
- *Time:* studies with at least 1 year of follow-up.

The exclusion criteria were lack of information about the sample size, deciduous or immature permanent teeth, animal or *in vitro* studies, case reports, literature reviews, editorials, and annals/presentations in professional meetings. 

### Searching strategy

The searching process was performed independently by three examiners (SCB, ARP, and EST). The PubMed database (all years up to May 2023) was electronically searched by using the MESH and entry terms and the Boolean operators AND and OR. Further search was performed through Embase, Web of Science, Scopus, and the Cochrane Library database. Gray literature was also consulted, through IBICT (Brazilian Institute of Information in Science and Technology)^,^ Brazilian Digital Library of Theses and Dissertations (BDTD) and Google Scholar. A search was also performed on the reference lists of all selected articles (Box1).

**Box:**
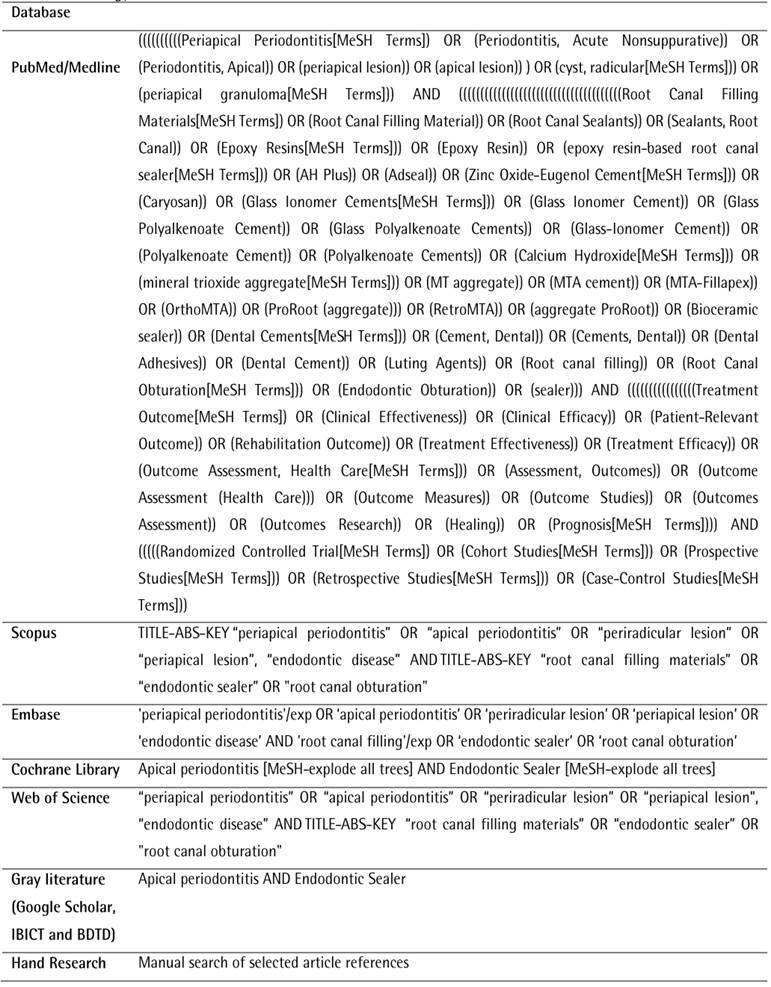
Search strategy.

The retrieved records were transferred to the EndNote Web® reference manager (EndNote web software, Clarivate Analytics, Philadelphia, PA, USA) to identify duplicates. A two-stage screening (titles and abstract first and then full-text) was carried out independently by two reviewers (PAS and SCB). When there was no agreement, a third examiner (ARP) was consulted and made the final decision. At the full-text stage, a data screening form was used to verify study eligibility, assess quality assessment and extract data on study characteristics and outcomes. The eligible studies were assessed for the outcome measures including ‘success rate’, radiographic/tomographic evidence of periradicular healing/reduction in lesion size, and clinical absence of signs and symptoms.

### Quality assessment (risk of bias)

Two reviewers (PAS and SCB) analyzed the risk of bias separately. Disagreements were resolved by consulting the third reviewer (ARP). The quality assessment for randomized control trials was performed according to the *Revised Cochrane risk of bias tool for randomized trials - ROB2* ^24^ ^),(^ ^25^ ^),(^ ^26^ This instrument is structured in five domains of bias: ^1^ randomization process, ^2^ deviations from the intended intervention, ^3^ missing outcome data, ^4^ outcome measurement, and ^5^ selection of the reported result. The judgments within each domain lead to an overall risk of bias judgment for the result being assessed. The judgment for each domain is "low risk of bias" (the study is judged to be at low risk of bias for all domains for this result^),^ "some concerns" (the study is judged to raise some concerns in at least one domain for this result, but not to be at high risk of bias for any domain^),^ or "high risk of bias" (the study is judged to be at high risk of bias in at least one domain for this result, or the study is judged to have some concerns for multiple domains in a way that substantially lowers confidence in the result). The overall risk of bias generally corresponds to the worst risk of bias in any of the domains. However, if a study is judged to have “some concerns” about risk of bias for multiple domains, it might be judged as at high risk of bias overall ^26^. 

Furthermore, the NOS scale *(Newcastle-Ottawa Scale)* ^28^ was used for prospective and retrospective cohort and case-control studies, by categorizing them into three dimensions: selection, comparability, and exposure/outcome. The selection, comparability, and exposure dimensions contain four, two, and three items, respectively. A star system was used as a semi-quantitative assessment of the study quality. A study received a maximum of one star for each numbered item within the selection and exposure categories. A maximum of two stars were awarded for comparability. The number of stars ranged from zero to nine (high-quality, > 7 stars; medium-quality, 4-6 stars; poor-quality, <4 stars) ^29^ ^).^

### Data extraction

The extracted data was also performed by two independent reviewers (PAS and SCB) and included: authors and year of publication, study design, number of patients/teeth/roots/canals, primary treatment/retreatment, instrumentation technique/final apical diameter, irrigation technique/solution, intracanal dressing, type of sealer, obturation technique, final restoration, obturation quality assessment, follow-up, evaluation method, outcome. 

## Results

**Figure 1:**
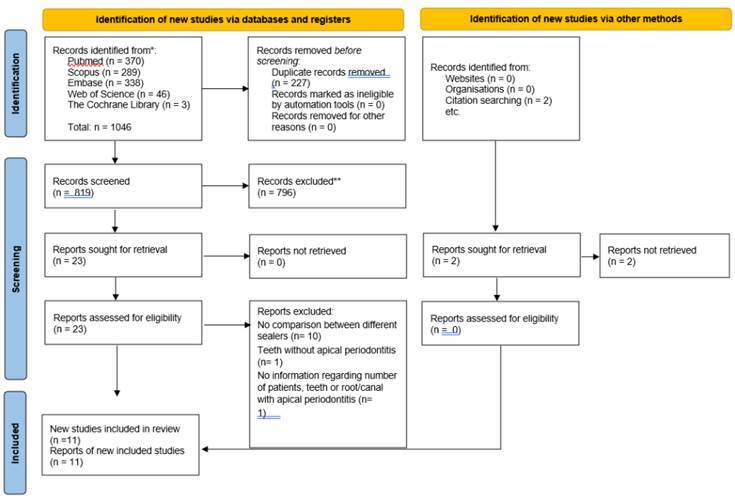
PRISMA flowchart with identification of the studies via databases and registers (PRISMA 2020 flow diagram).

**Figure 2:**
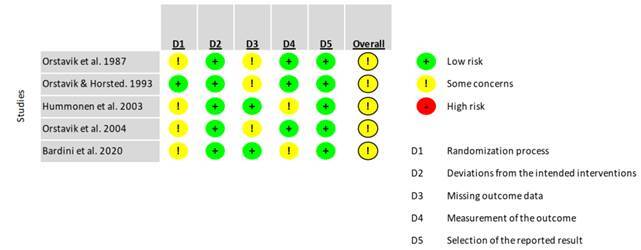
Risk of bias of the randomized clinical studies. (+) indicates a low risk of bias, (!) indicates some concerns, whilst (-) indicates a high risk of bias.

The results of the search strategy are shown in Figure 1. Database and hand search yielded 1,046 relevant references. After duplicate removal, 819 studies were screened by title and abstract, resulting in 23 potential articles for full-text review. Twelve were excluded as they did not completely meet the eligibility criteria (Box2): ten studies did not compare sealers ^30^ ^),(^ ^31^ ^),(^ ^32^ ^),(^ ^33^ ^),(^ ^4^ ^),(^ ^5^ ^),(^ ^36^ ^),(^ ^37^ ^),(^ ^3^ ^),(^ ^8^ ^),(^ ^39^ ^),^ one study assessed teeth without apical periodontitis ^40^ ^),^ and one study did not mention the number of patients, teeth, or root/root canal with apical periodontitis ^41^. The remaining 11 articles (1,467 patients/teeth with apical periodontitis) were included in the qualitative synthesis: 1 non-randomized clinical trial ^42^ ^),^ 5 randomized clinical trials ^43^ ^),(^ ^44^ ^),(^ ^45^ ^),(^ ^46^ ^),(^ ^47^ ^),^ 3 retrospective studies ^22^ ^),(^ ^48^ ^),(^ ^49^ ^),^ and 2 prospective cohort studies ^50^ ^),(^ ^51^. The quality assessment using RoB2 is described in figure 2 and included five randomized control trials studies ^43^ ^),(^ ^44^ ^),(^ ^45^ ^),(^ ^46^ ^),(^ ^47^. All the included randomized control trials received some concerns in the overall risk of bias.

The results of the NOS scale are shown in Box(3). The evaluated studies which showed ^22^ ^), (^ ^49^-^51^ and 8 stars ^48^ were classified with a low risk of bias. One study ^42^ had 5 stars and was considererd with medium risk of bias. The lack of stars for the above-mentioned studies was related to a lack of terms for the nonexposed cohort (related to study design), no description of the assessment outcome and follow-up rate <80%, without description of losses. The main extracted data are detailed in Box4. Supplementary data are presented in Box S1. A variety of missing analyses was observed, such as tooth type, number of roots/canals, lesion size, different instrumentation and obturation techniques.

**Box 2:**
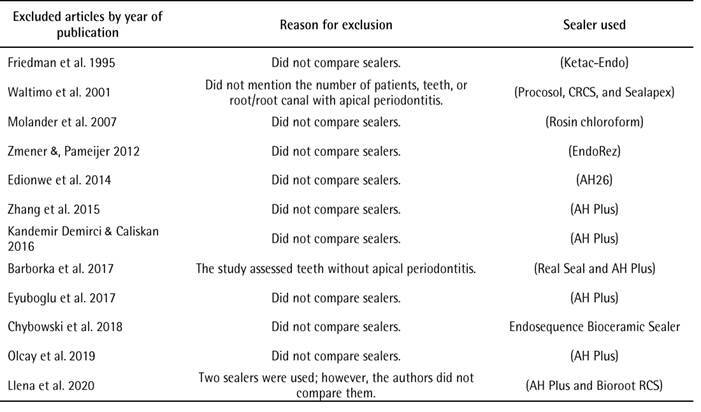
Excluded articles during full-text review.

**Box 3:**
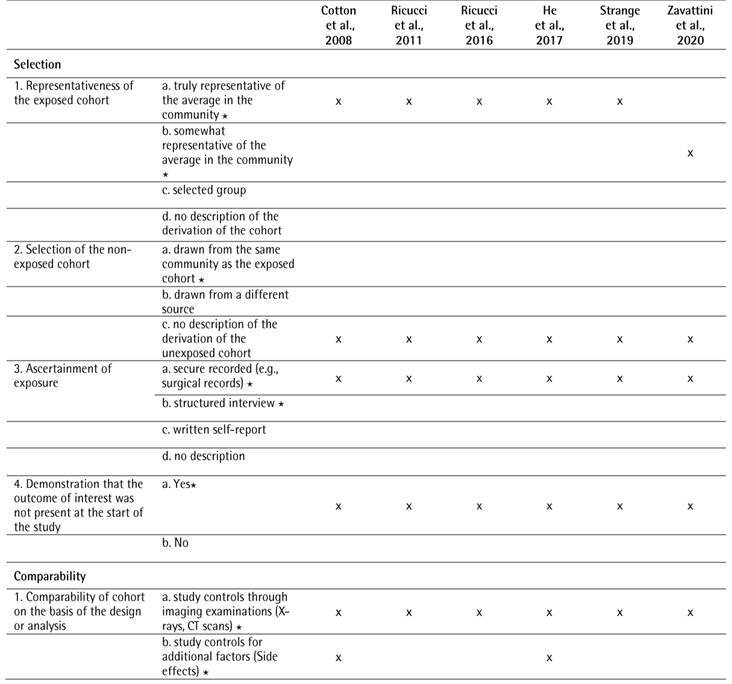
Quality analysis of the cohort studies according to the Newcastle-Ottawa Scale (NOS)

**Box 3:**
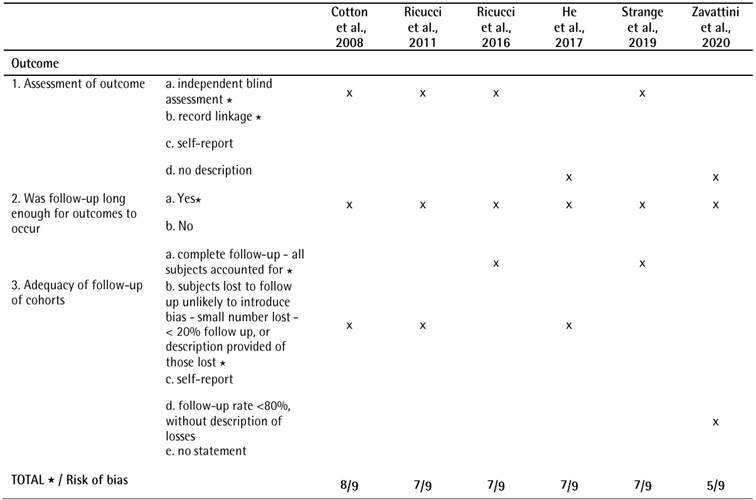
Continuation

A star system was used for allowing a semi quantitative assessment of study quality. A study was awarded a maximum of one star for each numbered item within the selection and exposure categories. A maximum of two stars were awarded for comparability. The NOS ranges from zero to nine stars. The number of stars ranged from zero to nine (high-quality, ³7 stars; medium-quality, 4-6 stars; poor-quality, < 4 stars).

The selected articles have not specified which tooth types had apical periodontitis. Only two studies ^45^ ^),(^ ^47^ specified the gender of the patients, totaling 243 patients (136 females and 107 males). However, no further detail of the number of teeth or roots/root canals with apical periodontitis was reported. Only one study ^45^ had 100% of the patients (n=199) with apical periodontitis. Nine studies reported the number of treated teeth with apical periodontitis ^42^ ^), (^ ^44^ ^), (^ ^46^-^52^ ^),^ which ranged from 45 to 285, totaling 951 teeth. One study reported just the number of treated roots with radiographic periapical lesion (233 roots with periapical index (PAI) scores 3, 4 and 5) ^43^ and one ^46^ reported the number of roots with apical periodontitis, dividing them as single or multiple roots. Finally, regarding the number of root canals, one study reported that 15 single canals and 50 multiple canals had apical periodontitis ^48^ ^),^ and other reported 359 root canals with apical periodontitis (50).

### Root canal treatment or retreatment 

Six studies performed primary root canal treatment ^42^ ^), (^ ^43^ ^), (^ ^45^ ^), (^ ^46^ ^), (^ ^49^ ^), (^ ^52^ ^),^ while four studies performed both treatment and retreatment ^44^ ^), (^ ^47^ ^), (^ ^48^ ^), (^ ^50^. One study ^51^ performed only retreatments. Root canal preparation was performed with several manual or rotary instrumentation and apical sizes varied from 25 to 40.

### Root canal irrigation and intracanal medication

A variety of information regarding root canal irrigation and intracanal dressing between appointments was found among the studies, many of which missing a detailed description. Irrigant solutions included 0.5%, 1%, 2%, 5% and 5.25% sodium hypochlorite (NaOCl) and 5% chloramine-T; 17% EDTA and hydrogen peroxide were also mentioned. Syringe needle irrigation was the most used approach, while the use of passive ultrasonic irrigation and sonic activation of irrigant with the EndoActivator device was reported as supplementary irrigation methods in two studies ^42^ ^),(^ ^43^ ^),(^ ^44^ ^),(^ ^45^ ^),(^ ^46^ ^),(^ ^47^ ^),(^ ^48^ ^),(^ ^49^ ^),(^ ^50^ ^),(^ ^51^.

**Box 4:**
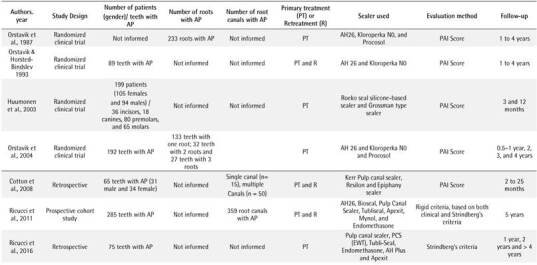
Data extracted from the selected studies.

**Box 4:**
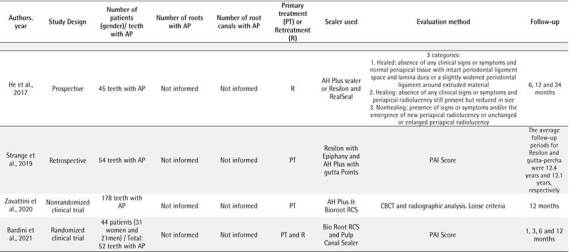
Continuation.

AP: Apical periodontitis; PAI: periapical index

Regarding interappointment intracanal medication, calcium hydroxide was the most used substance, while iodoform or metacresyl acetate applied on a cotton pellet were also reported. However, some studies failed to specify the type of medication used between appointments or if it was used at all ^42^ ^),(^ ^43^ ^),(^ ^44^ ^),(^ ^47^ ^),(^ ^49^.

### Type of sealers and obturation techniques

The type of sealers used for root canal obturation varied from zinc oxide-eugenol-based ^43^ ^), (^ ^46^-^48^ ^),(^ ^50^ ^),(^ ^52^ ^),^ epoxy resin-based ^42^ ^),(^ ^43^ ^),(^ ^44^ ^),(^ ^46^ ^),(^ ^49^ ^),(^ ^50^ ^),(^ ^51^ ^),(^ ^52^ ^),^ calcium hydroxide-containing ^50^ ^),(^ ^52^ ^),^ bioceramic ^42^ ^),(^ ^47^ ^),^ chloroform-gutta-percha ^43^ ^),(^ ^44^ ^),(^ ^46^ ^),^ silicone-based ^45^ ^),^ and methacrylate sealers^51^ ^),^ and dual-cure dental resin composite sealer with thermoplastic synthetic polymer-based solid material ^48^ ^), (^ ^49^. Diverse obturation techniques were used, including lateral compaction ^43^ ^),(^ ^44^ ^),(^ ^45^ ^),(^ ^46^ ^),(^ ^50^ ^),(^ ^52^ ^),^ continuous wave of compaction ^48^ ^), (^ ^51^ ^),^ single cone technique ^42^ ^),(^ ^47^ and warm vertical compaction ^42^ ^),(^ ^47^. One study did not mention the obturation technique used ^49^. Regarding funding sources, only two studies reported that they received support from industry ^45^ ^),(^ ^42^. 

Not all authors informed the type of final restoration placed after root canal treatment ^43^ ^),(^ ^44^ ^),(^ ^46^ ^),(^ ^49^ ^),(^ ^50^ ^),^ while others varied from direct composite as permanent build up ^42^ ^), (^ ^47^ ^), (^ ^48^ ^), (^ ^51^ ^), (^ ^52^ ^),^ full cusp coverage ^42^ ^),^ indirect restoration with full cusp coverage 52 or amalgam 48, and provisional materials such as IRM or Cavit ^48^ ^), (^ ^51^.

### Clinical and Radiographic Follow-up

Clinical and radiographic follow-up periods ranged from 1 to 12.4 years ^47^ ^), (^ ^49^ with a mean overall follow-up time of 3.7 years. To evaluate the outcome of root canal treatment, most studies ^43^-^49^ used the 2-dimensional radiographic PAI score method ^53^ ^),^ while others ^50^ ^), (^ ^52^ used the Strindberg’s criteria ^54^. Another study classified the treatment outcomes by defining 3 categories based on digital periapical radiographs: healed, healing and nonhealing ^51^. Finally, only one recent study ^42^ used cone-beam computed tomography (CBCT) along with periapical radiographs for an outcome analysis defined by loose criteria (reduction in lesion size) ^55^. 

### Outcomes

Eight studies found no significant differences when comparing sealers ^42^ ^), (^ ^44^ ^), (^ ^46^-^48^ ^), (^ ^51^ ^), (^ ^52^ (Box5). The healing rates ranged from 56.7% ^47^ to 90% ^51^. Ricucci *et al.* ^52^ ^),^ compared six types of sealers, but evaluated only cases of unintentional overfillings. They found no statistically significant difference in the outcome between the sealers. Relatively new endodontic sealers (bioceramics) were compared in two recent clinical studies ^42^ ^), (^ ^47^. The success rates for BioRoot RCS and Pulp Canal Sealer (PCS) were 76.9% and 56.7%, respectively; this difference however was not statistically significant (p>0.05) ^47^. Another study found that BioRoot RCS in combination with single cone resulted in a success rate comparable to that of warm vertical condensation and AH Plus sealer (p>0.05).

The remainder of the studies found different responses to periapical healing after endodontic treatment when different sealers were used (Box 5). A study found worsening of periapical healing after one year when Kloroperka was used, which was even more significant (p<0.05) after the second and third years of follow-up, in comparison with AH 26 and Procosol sealer ^(43)^. However, it is important to point out that Kloroperka is not a sealer per se. Additionally, according to Ricucci *et al.* ^50^ ^),^ AH26, Bioseal, Mynol, and Apexit sealers had higher success rates at 5 years follow-up than PCS, Endomethasone, and Tubliseal in teeth with apical periodontitis. The success rate was also influenced by the size of radiographic lesion, and if it was treatment or retreatment. A long-term outcome study (12 years follow-up) showed that subjects with Resilon/Epiphany treated teeth were more likely to have an apical periodontitis lesion than teeth filled with gutta-percha/AH Plus ^49^. Moreover, the adjusted odds ratio showed that teeth obturated with Resilon/Epiphany were 5.3 times more likely to have a lesion at follow-up than those treated with gutta-percha/AH Plus. 

**Box 5:**
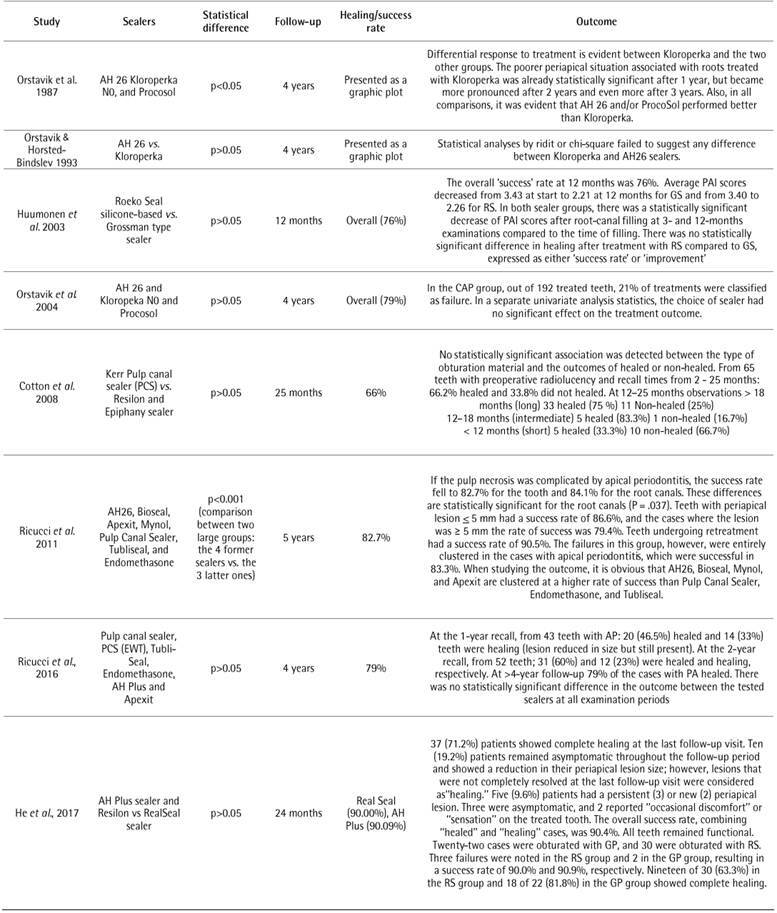
Outcome of root canal treatment when two or more sealers were compared.

**Box 5:**
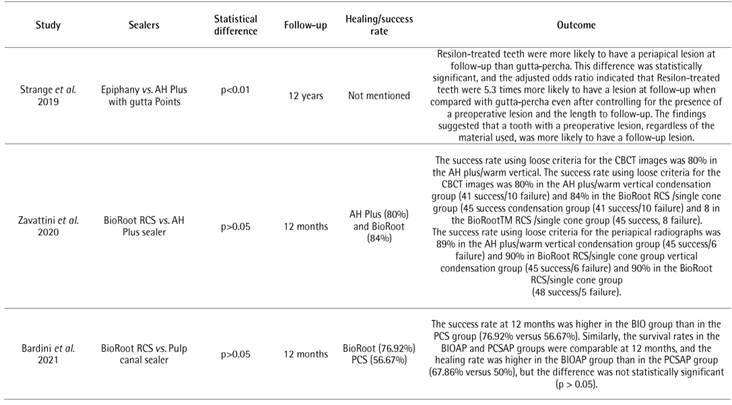
Continuation

## Discussion

This systematic review focused on the question of whether the type of root canal sealer can influence the outcome of the nonsurgical root canal treatment or retreatment of teeth with apical periodontitis. A recent systematic review 7 investigated if obturation techniques and materials used for root canal filling influenced the treatment of apical periodontitis. The authors found very heterogeneous data and a high risk of bias of the included studies. As for sealers, the previous review was limited to studies comparing 2 sealers (AH Plus and AH26) with any other; as such, only two studies were included for the analysis of sealers. The present review in turn focused on any comparisons involving sealers, which resulted in the selection of 11 studies, most of which with a medium risk of bias. Findings from both studies indicate that the treatment outcome is not affected by sealers.

One noBoxexception is the Resilon/Epiphany (Resilon Research LLC, Madison, CT). Although a preliminary short-term study (1 to 2-year follow-up) reported comparable results between Resilon/Epiphany and gutta-percha/Kerr Pulp Canal Sealer (Kerr corporation, Orange, CA, USA) (Cotton et al., 2008^),^ long-term data (> 5 years) revealed a significantly worse outcome in the former ^(40, 49).^ Barborka et al. ^40^ observed that 56% of the teeth filled with Resilon/Real Seal SE were successful as compared to 88% of the gutta-percha-AH Plus-filled teeth. In another study ^49^ ^),^ teeth filled with Resilon/Epiphany exhibited 5.3 times higher prevalence of apical periodontitis when compared with gutta-percha/AH Plus. The reasons for the worst results presented by Resilon/Epiphany may be related to enzymatic degradation of the material and creation of a gap between the sealer and the core ^56^ ^),^ failure to resist shrinkage stress ^57^ ^),^ and high cytotoxicity ^58^. 

Apart from the old data on Kloroperka (a filling material based on gutta-percha softened in chloroform) and the most recent ones on Resilon/Epiphany, there is only one cohort study showing significant different outcomes according to the sealer type. In a prospective cohort study, Ricucci *et al.* ^50^ found that collectively the sealers AH26, BioSeal, Mynol, and Apexit, all with more than 92% success rate, had better outcomes at 5-year follow-up when compared to PCS, Endomethasone, and Tubliseal, with less than 88% success. However, the sealers were included in two groups for statistical analysis. We used the data available in the article for a pairwise comparison between sealers using the chi-square test with Yates correction and the only differences observed were for PCS compared with Apexit or Bioseal (p<0.05). Moreover, no distinction was made in the article as for the periradicular status before treatments. Therefore, it is not possible to ascertain the influence of these sealers in teeth with apical periodontitis only, which is the purpose of this systematic review. 

All the other studies included showed that the root canal sealer does not exert a significant influence on the outcome of the root canal treatment of teeth with apical periodontitis. This is in agreement with a previous systematic review of diverse clinical factors with potential to influence the treatment outcome ^59^. 

Some relatively new materials, including calcium silicate-based or bioceramic sealers, have not been used in clinical practice for sufficient time to be assessed in long-term outcome studies. Consequently, there is limited information about their influence on treatment outcome. Calcium silicate-based sealers have shown promising physicochemical and biologic properties in comparison with conventional sealers ^60^ ^-^ ^62^ ^).^ However, the few outcome studies published so far show no improved results in comparison with conventional materials ^42^ ^,^ ^47^.

A study evaluating the fate and influence on the treatment outcome of different sealers that were accidentally extruded to the periapical tissues demonstrated that, while there were differences in the removal rate of the different materials over time, none of them was shown to influence the outcome of teeth with or without apical periodontitis ^52^. Teeth with preoperative apical periodontitis showed a worse outcome, but with no difference between the different materials. The main causes of a compromised outcome in teeth with extruded material overfilling have been suggested to be related to an inadequate apical seal or previous overinstrumentation, and not the effects of the material itself ^63^ ^).^ In spite of being exclusively focused on the outcome of overfillings, that study was included in the systematic review because it represented the extreme of contact area between the filling material and the periradicular tissues. 

Unlike several cytotoxic filling materials used in the past, the large majority of current materials may exhibit some cytotoxicity before setting ^64^ ^-^ ^66^. As demonstrated in many short-term animal studies ^67^ ^-^ ^69^ ^),^ these materials can be associated with an inflammatory response of varying intensity in the periodontal ligament tissue area in contact with the material. In overfillings, the material volume and the contact area are larger, and consequently the inflammatory response is more severe ^70^. However, because most filling materials lose their toxicity with the passage of time ^71^ ^,^ ^72^ ^),^ the tissue aggression is not sufficient to cause or sustain an apical periodontitis lesion. Therefore, in the absence of concurrent residual infection, most currently available filling materials per se may not influence the outcome of the endodontic treatment, as demonstrated by most studies reviewed herein. Despite a few reports in the literature ^19^ ^,^ ^73^ ^),^ the present findings also suggest that most current filling materials do not cause a foreign-body reaction, and as such are not the cause of posttreatment apical periodontitis.

Many properties of root canal filling materials might potentially interfere with the treatment outcome, especially cytotoxicity, sealing ability, and antibacterial activity. If the material is too toxic to human cells, it can cause tissue damage and lead to or maintain inflammation. Materials that fail to promote an adequate seal permit seepage of inflammatory exudate or tissue fluid into the canal, providing residual bacteria with nutrients to grow and cause or maintain apical periodontitis ^74^. Materials with antibacterial properties may contribute to additional disinfection by killing residual bacteria ^16^ ^,^ ^75^. These properties are influenced by many others including adhesion to dentin, solubility, dimensional stability, and flow ability. The literature is plenty with *in vitro, ex vivo* and animal studies evaluating the properties of endodontic filling materials ^62^ ^,^ ^76^ ^,^ ^77^ ^,^ ^79^. Despite the differences usually reported in most of these studies for different sealers, this systematic review suggests that such differences may not suffice to represent benefits in terms of improved outcome for any of the sealers currently available. The treatment steps involved with infection control before obturation are expected to have more impact on the treatment outcome ^9^ ^,^ ^10^.

The present study has not evaluated if the type of material can influence the healing time. Some studies have reported longer healing times for some overfilled teeth (52,82^),^ but whether the reasons for that are the filling material type, concomitant low-grade infection and/or host-related factors remain to be elucidated. 

This systematic review has certainly limitations. The main shortcoming is that there is a remarkable scarcity of prospective studies about the long-term management and outcome of patients with apical periodontitis. This is especially true for randomized controlled clinical trials. The heterogeneity of the selected articles was evident right from the beginning. Also, a variety of missing analyses was observed, such as tooth type, number of roots/canals, lesion size, different instrumentation and obturation techniques. In context, it was not possible to conduct a meta-analysis because the data were widely dispersed, and the studies were very heterogeneous, with several methodological differences. Moreover, subgroup analysis (gender; age; size of the lesion; type of tooth; number of root canals) could not be performed in this review due to the lack of data from the selected studies. In addition, it is known that there are many variables that can influence the treatment outcome, such as the instrumentation technique and the irrigants used, tooth type, and host-related factors (age, systemic condition, etc.).

Based on this systematic review of clinical outcome studies, the current endodontic sealers do not seem to influence the treatment outcome of teeth with apical periodontitis. Although the studies were of medium and low risk of bias, the results should be interpreted with caution. Further randomized long-term outcome studies comparing filling materials are required to strengthen this assertion and permit a meta-analysis to be conducted. Despite differences between studies for different sealers, this systematic review suggests that such differences may not be sufficient to represent benefits in terms of better outcome for any of the currently available sealers.

## Figures and Tables

**Box S1:**
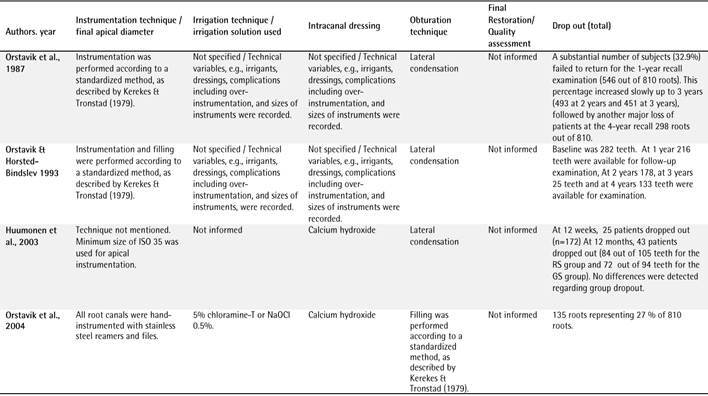
Supplementary data of the selected studies.

**Box S1:**
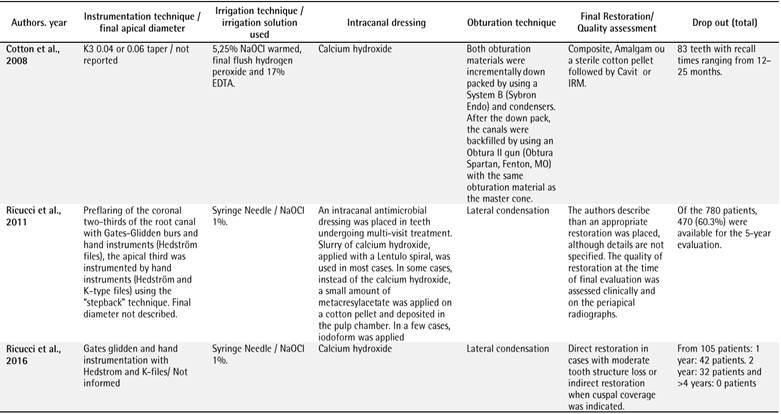
Continuation

**Box S1:**
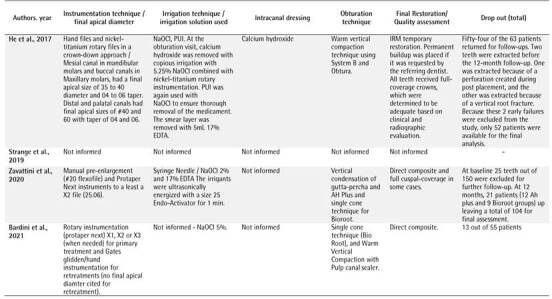
Continuation
